# Association between physical function and perceived stress among U.S. Chinese older adults

**DOI:** 10.46439/aging.1.004

**Published:** 2020

**Authors:** Ying-Yu Chao, Peijia Zha, Kyeongra Yang, XinQi Dong

**Affiliations:** 1 Clinical Assistant Professor, Rutgers, the State University of New Jersey, School of Nursing, 180 University Avenue, Newark, NJ 07102-1803, USA; 2 Assistant Professor, Rutgers, the State University of New Jersey, School of Nursing, 180 University Avenue, Newark, NJ 07102-1803, USA; 3 Associate Professor, Rutgers, the State University of New Jersey, School of Nursing, 65 Bergen Street, Room 1025E, Newark, NJ 07107, USA; 4 Director, Rutgers Institute for Health, Health Care Policy and Aging Research, 112 Paterson St, New Brunswick, NJ, 08901, USA

**Keywords:** Physical function, Perceived stress, Chinese, Older adults, Immigrants

## Abstract

**Objectives:**

Physical function impairment can cause great stress to older adults. The purpose of the study is to investigate the association between self-reported and directly-observed physical function on perceived stress among U.S. Chinese older adults.

**Methods:**

Data were from the Population Study of Chinese Elderly in Chicago (PINE) of 3,157 Chinese older adults who were 60 and above in the Greater Chicago Area. Self-reported and directly-observed physical function measures, and Perceived Stress Scale were used.

**Results:**

Participants had a mean age of 72.8 ± 8.3 years old (range 60–105). Higher scores of Katz activities of daily living impairments (odds ratio [OR]=1.77), Lawton instrumental activities of daily living impartments (OR=1.10, *p*<0.01), Rosow–Breslau index of mobility scale (OR=1.39, *p*<0.05), and Nagi index of basic physical activities scale (OR=1.19, *p*<0.001) were associated with higher levels of perceived stress. In addition, higher scores of directly-observed physical function measurements, including chair stand (OR=0.93), tandem stand (OR=0.71, *p*<0.05), timed walk (OR=0.73, *p*<0.001), and the overall measurement (OR= 0.87, *p*<0.01) were associated with lower level of perceived stress.

**Discussion:**

Findings suggested that poor physical function was associated with perceived stress among U.S. Chinese older adults. Longitudinal studies are needed to obtain a more comprehensive understanding of the pathways between physical function and perceived stress.

**Implications for practice:**

Health care professionals could provide personalized physical activity interventions to encourage older adults to engage in regular exercise in order to maintain and promote older adults’ physical function and psychological well-being.

## Introduction

Chinese Americans are the oldest and largest Asian population in the United States. Approximately 16% of the 4.4 million U.S. Chinese population were over age 65. More than 80% of Chinese older adults residing in the U.S. were foreign-born, and about 30% of them immigrated to the U.S. at the age of 60 or over [1]. Immigration is a highly stressful process. During the acculturative process, Chinese older immigrants experienced greater psychological distress and health disparities [2]. According to a transactional stress model [3], perceived stress represents the psychological perception of environmental demands exceeding personal coping skills and resources. Perceived stress among Chinese older immigrants has been attributed to the loss of own social environment, discrimination, language barriers, conflict with the dominant American culture, and lack of social support [4]. In addition, compared with younger age groups, older adults may experience more stress as they age due to loneliness, bereavement, caring for a sick spouse, and deterioration of their own health [5,6].

Physical function is critical to maintaining independence and social interactions in later life [7]. Individuals with physical disabilities may be more vulnerable to stress and have few resources to cope with stress, resulting in higher levels of stress. The major underlying causes of physical disability can be acute events (e.g., hip fracture and stroke), and chronic disease (e.g., arthritis, congestive heart failure, and diabetes) [7]. The common risk factors for lower levels of physical function include lower socioeconomic status, older age, lower education level, and worsening health status [8,9]. Poor physical function has shown to increase the risk of disability, morbidity, hospitalizations, nursing home admissions, and mortality [7,9]. Researchers proposed that findings of self-reported and directly-observed measures in physical function may not be strongly associated due to self-reported biases, and the measures may not measure the same construct [10]. Combining both measures may decrease the potential biases as well as provide more objective and uniform results. In other words, using both self-reported and directly-observed measures can complement each other in providing comprehensive information about the physical function status of older adults [9].

There is growing evidence that older Asian Americans have a higher prevalence of physical functional impairments and disabilities in comparison with their U.S.-born counterparts [9]. Because of the various socioeconomic status, cultural beliefs, health literacy, and available social services, U.S. Chinese older adults may disproportionately experience physical functional decline compared with U.S. general older adults [11]. However, many studies examine Asian Americans as an aggregate group, which make it difficult to understand the ethnic group differences and diverse experiences [12]. As Chinese older adults are the fastest growing segment of the aging population in the U.S. [13], it is important to understand the association between physical function and perceived stress among this group. However, to the best of our knowledge, the associated issue of a better understanding of how both self-reported and directly-observed aspects of physical impairment affect perceived stress in U.S. Chinese older adults is an important avenue of investigation that has not been thoroughly treated in this research area. Hence, the purpose of the present study is to investigate the association between self-reported and directly-observed physical function on the perceived stress among U.S. Chinese older adults. The specific aims are to: (1) examine whether self-reported and directly-observed physical function differed by perceived stress; (2) examine the unique contributions of self-reported and directly-observed physical function on the perceived stress.

## Methods

### Population and setting

The Population Study of Chinese Elderly in Chicago (PINE) is a community-engaged, population-based epidemiological study. The PINE study was conducted to examine key cultural determinants of health and well-being in community-dwelling Chinese older adults in the Greater Chicago Area. The project was initiated by a synergistic community-academic collaboration among Rush Institute for Healthy Aging, Northwestern University, and over 20 community-based social services agencies and organizations throughout the Greater Chicago Area. The PINE study implemented extensive culturally and linguistically appropriate recruitment strategies through a community-based participatory research approach. The inclusion criteria of participants included: (1) older adults over the age of 60 years old, (2) self-identified as Chinese; (3) resided in the Greater Chicago Area. Details of the PINE study design were published elsewhere [1]. Data in this paper were drawn from the first-wave PINE study that was collected between 2011 to 2013. Eligible participants were 3,542 Chinese older adults. The response rate was 91.9% (N=3,159) [13].

### Measurements

#### Independent variables: Physical function

Self-reported physical function was assessed with four well-established measures. The first was Katz activities of daily living (ADL), which measured physical function impairment in participants’ ability to perform basic self-care tasks, such as dressing, bathing, toileting, etc. [14]. The second measure was the Lawton instrumental activities of daily living (IADL), which asked participants to self-report the extent of help needed in 12 different activities, such as taking medication, prepare meals, shopping, etc. [15]. The third measure was a Rosow–Breslau index of mobility scale, which asked participants’ abilities to perform physical tasks, such as doing heavy work around the house, and walking a half mile without help [16]. The fourth measure was Nagi index of basic physical activities scale, measuring five activities of upper and lower extremity function [17]. Higher scores of ADL impairment, IADL impairment, index of mobility scale, and index of basic physical activities scale indicated higher levels of physical function impairment. In the PINE sample, ADL (Cronbach’s alpha=0.92), IADL (Cronbach’s alpha=0.90), Index of Mobility scale (Cronbach’s alpha=0.80), and Index of Basic Physical Activities scale (Cronbach’s alpha=0.80) had good validity and internal consistency [9].

As for the directly-observed physical function testing, the Short Physical Performance Battery (SPPB) was used. The first test was the chair stand, measuring participants’ ability to rise to a standing position from a chair. The second test was the tandem stand, which asked participants to perform semi-tandem, then full tandem or side-by-side (depending on the performance on semi-tandem). The third test was an 8-foot timed walk. Higher scores of physical performance testing indicated better functional performance. The SPPB in the PINE sample had good validity and inter-rater reliability (Cronbach’s alpha=0.69) [9].

#### Dependent variable: Perceived stress

Perceived stress was assessed with the 10-item Perceived Stress Scale (PSS-10) [18]. Participants were asked the degree to which situations in lives were perceived as stressful in last month, including (1) upset because something happened unexpectedly; (2) unable to control important things in life; (3) nervous and stressed; (4) confident about the ability to handle personal problems; (5) things were going your way; (6) could not cope with all the things had to be done; (7) been able to control irritations in life; (8) were on top of things; (9) angered because of things that happened not in their control; and (10) could not overcome piled up difficulties. Respondents of each item on a 5-point scale, ranging from 0 (never) to 4 (very often). Of the 10 items, 4 items (items 4,5,7,8) were reverse-scored. The total score ranged from 0 to 40, with higher scores indicating greater psychological stress [18]. The Chinese PSS had acceptable validity and internal reliability (Cronbach’s alpha=0.67–0.83) in the previous studies [19,20]. The validity and reliability of Chinese PSS-10 in the PINE study were excellent (Cronbach’s alpha=0.86) [4]. We first categorized participants as “no stress” versus “any stress”.

#### Confounding factors

Social-demographic characteristics included age, gender, years of education completed (0–8, 9–12, 13 and above), annual personal income ($0–4,999; $5,000–9,999 $10,000–14,999; $15,000 and more), living arrangement (alone, with 1–2 person, with = 3 persons), years in the community (0–9, 10–19, 20–29, 30 and more), years in the United States (0–9, 10–19, 20–29, 30 and more), and medical comorbidities. The number of medical comorbidities was calculated by summing the number of “yes” responses to the nine groups of the following medical conditions: (1) heart disease; (2) stroke or brain hemorrhage; (3) cancer, malignancy or tumor of any type; (4) high cholesterol; (5) diabetes; (6) high blood pressure; (7) a broken or fractured hip; (8) thyroid disease; and (9) osteoarthritis, inflammation, or problems with joints Confounding factors were chosen and categorized based on the previous studies [4,9].

### Data analysis

Chi-square and t-test were used to compare the mean differences in socio-demographics and physical function between participants with and without perceived stress. Spearman correlations were used to measure the correlation between perceived stress and physical function. Multivariate logistic regression models were used to examine the association between physical function and perceived stress. Potential covariates were controlled in the models. Model A was adjusted for basic socio-demographic characteristics, including age, gender, education. Income and living arrangement were added to Model B. In Model C, years in the community and years living in the United States were included in addition to Model B. We then added medical comorbidities to build Model D. Odds ratios (ORs), 95% confidence intervals (CIs), and significance levels were reported. All statistical analyses were conducted using SAS, Version 9.2 (SAS Institute Inc., Cary, North Carolina).

## Results

This study of Chinese older adults used a total of 3,157 participants with a mean age of 72.8 years (SD=8.3, range 60–105). Of the participants in the study, 58.9% were female, while 71.3% were married. 78.9% had less the equivalent of high school education, or less. Most of them (85.1%) had an annual income less than US$10K. More than half of the participants (55.6%) had three or more children. 21% of the participants lived alone. 73.3% of the participants had resided in the U.S. for more than 10 years. A total of 96.08% of study participants perceived certain levels of stress. Table 1 is the characteristics of study participants with and without perceived stress. Compared with older adults reported no stress, those reported perceiving stress were more likely to be an older age (*p*<.001), female (*p*<0.05), have lower income (*p*<0.01), fewer household members (*p*<0.01), fewer years in the community (*p*<0.05), and more medical comorbidities (*p*<0.05). In terms of physical function, older adults with perceived stress were more likely to have higher scores in ADL impairment (*p*<0.05), IADL impairment (*p*<0.001), index of mobility scale (*p*<0.001), index of basic physical activities scale (*p*<0.001), chair stand (*p*<0.01), tandem stand (*p*<0.001), timed walk (*p*<0.001), and SPPB (*p*<0.001) compared with those without perceived stress, indicating that older adults with perceived stress were more likely to have poorer self-reported and directly-observed physical function. Table 2 shows the correlation between perceived stress and physical function. Perceived stress was significantly correlated with ADL impairment, IADL impairment, index of mobility scale, index of basic physical activities scale, chair stand, tandem stand, timed walk, and SPPB. The correlation coefficients ranged from −0.27 to 0.34.

### Associations between physical function and perceived stress

Table 3 shows the associations between self-reported physical function and perceived stress. After adjusting all confounding factors, although not significant, Katz ADL impairments were associated with perceived stress (OR=1.77, 95% CI=0.78–3.99). Chinese older adults with a higher score of Lawton IADL impartments were 10% (OR=1.10, 95% CI=1.03–1.18, *p*<0.01) significantly more likely to report perceived stress, compared to those reported no stress. Chinese older adults with a higher score of index of mobility scale were 39% (OR=1.39, 95% CI=1.07–1.82, *p*<0.05) significantly more likely to report perceived stress, compared to reporting no stress. Chinese older adults with a higher score of index of basic physical activities scale were 19% (OR=1.19, 95% CI=1.09–1.30, *p*<0.001) significantly more likely to report perceived stress, compared with those who reported having no perceived stress.

Table 4 shows the associations between directly-observed physical function and perceived stress. In the fully adjusted model, after controlling for confounding factors, the directly-observed physical function measurements, except chair stand, were significantly associated with perceived stress (tandem stand: OR=0.71, 95% CI=0.52–0.96, *p*<0.05; timed walk: OR=0.73, 95% CI=0.63–0.86, *p*<0.001; and SPPB: OR=0.87, 95% CI=0.80–0.95, *p*<0.01). Every one-point increases in the tandem stand, timed walk, and SPPB were associated with 29%, 27%, and 13% decreased odds of reporting perceived stress, respectively.

## Discussion

This is the first and largest population-based epidemiological study to investigate the association between physical function and perceived stress among U.S. Chinese older adults in the Greater Chicago Area. Our study found that older adults who had perceived stress were more likely to be an older age, female, have lower income, fewer household members, and fewer years in the community compared with those who reported having no perceived stress. Our study indicates that stress levels were higher among participants with an older age as older age is associated with declined in physical function, and increased risk of losing significant others and life partners [4,21]. With respect to gender, our female participants had higher stress levels than male participants. It is possible that females tend to have longer life expectancies and therefore more likely to be widowed, live alone, and experience feelings of loss [4]. In addition, the majority of our study participants had never employed in U.S. job market [22]. Having low income exacerbated the negative emotional well-being associated with illness [23]. The financial hardships (e.g., lacking social security and employment pensions) may lead to higher levels of perceived stress. Moreover, fewer household members and fewer years in the community may increase the risks of exposure to stressors. It is possible that older immigrants may have less emotional support and limited social networks in U.S society caused by linguistic and cultural barriers [4]. Furthermore, our participants who had more medical comorbidities had perceived more stress. It is possible that Chinese older adults with a greater number of medical comorbidities may experience a faster decline in physical function and poor physical health [4,6].

Our study demonstrated that both self-reported and directly-observed physical function were associated with perceived stress among Chinese older adults. We found that U.S. Chinese older adults with poorer self-reported IADL impairment, index of mobility scale, and index of basic physical activities scale were more likely to perceive higher levels of stress compared with those with better self-reported physical function. When older adults cannot take care of their basic needs (i.e., bathing, feeding, dressing, and toilet hygiene) and have difficulty going out shopping alone, or going to a medical appointment, they may feel incapable and dependent, and thus feel distressed and pessimistic [24]. Our results supported Kulmala et al. [25] study that individuals with more severe ADL and IADL disabilities had more constant stress symptoms than those with less disability. Our findings also supported a population-based epidemiological study that older adults having fewer problems performing IADL had lesser psychological distress [26].

With respect to directly-observed measures, our participants with better functional performance on tandem stand, timed walk, and SPPB were less likely to perceive stress compared with those with poorer physical performance. Our results supported Bellon et al. [27] study that individuals with traumatic brain injury having better mobility had significantly decreased perceived stress after walking intervention [27]. Similar to the previous finding [25], mobility limitation was associated with perceived stress. However, perceived stress may be varied in different ethnic/racial groups. For example, perceived stress was associated with greater osteoarthritis pain during balance, walking, and chair stand tasks among non-Hispanic Blacks, but not among non-Hispanic Whites [28]. Early research identified discordant results might exist between self-reported and directly-observed measures in physical function [10]. The differences of study findings may be due to the inconsistency of variable definitions, data collection processes, assessment instruments, and other variations across studies [9]. Nevertheless, our study showed that the poor self-reported and directly-observed physical function were associated with perceived stress among Chinese older adults in the Greater Chicago Area.

Supporting the transactional model of stress [3], the present study suggests that physical function impairment could profoundly influence perceived stress in older Chinese-Americans. Physical function impairment could increase stress at various levels, including compromised social engagement for individuals [13], increased caregiving burden for families [29], and healthcare costs [21]. The stress can influence an individual’s health, and unmanaged stress could lead to or exacerbate metabolic disease (e.g., diabetes), psychiatric disease (e.g., depression), sleep disorders, cardiovascular disease, cognitive function impairment, and immune dysfunction [30,31]. Physical activity has shown to ameliorate the progressive decline in physical function. It is well known that resistance exercise (e.g., strength training) can improve muscle mass, strength, functional independence, physical performance, and walking speed [32]. Aerobic exercise (e.g., walking) has also shown to improve gait and walking speed [33]. Tai Chi practice can improve physical function and reduce falls in older adults [34]. As such, health care professionals could provide older adults with personalized physical activity interventions at preventing the decline of physical functioning and promoting healthy aging.

Chinese culture has been enforcing filial piety as an essential moral standard [11]. Chinese older adults may have a strong sense of filial piety and the expectation that their adult children should take care of them when they were ill or disabled. Older adults with poor physical function may not only worry about their own health but also worry about adult children’s behaviors in relation to expectations of filial piety. Adult children’s neglect may violate the older adult’s expectation of personalized care and may further enhance the level of personal perceived stress. Therefore, Chinese adult children are encouraged to pay more attention to take care of their older parents with physical function impairment to avoid older parents perceiving children not performing caregiving responsibilities. On the other hand, due to cultural influence, Chinese adult children may often ask frail older adults to lay down and rest, instead of doing exercise, which may further exacerbate physical decline. Hence, it is important to balance filial piety obligations and parents’ physical needs of regular exercise to prevent physical function decline.

The study was presented with several limitations. First, this study was representative of Chinese older adults in the Greater Chicago Area. Hence, the finding may not be generalizable to Chinese older adults in other geographic areas. Second, this was a cross-sectional study, so we did not know the causal directions of physical function and perceived stress. Futures studies with longitudinal designs are needed to obtain a more comprehensive understanding of the pathways between physical function and perceived stress among U.S. Chinese older adults. Third, some factors (e.g., acculturation, filial piety, and social support) may influence the perceived stress on older Chinese immigrants. Future studies could include potential risk or protective factors to examine possible mediating or moderating effects between physical function on perceived stress.

Despite the limitations, our findings have important implications. For research implications, our results showed that poor physical function was associated with perceived stress among U.S. Chinese older adults. This study allowed us to test whether the physical function was associated with perceived stress and whether findings differed when using a self-reported measure of physical function compared with a directly-observed measure of physical function. The results lay the groundwork for research investigating the longitudinal relationship between physical function and perceived stress in U.S. Chinese older adults. In addition, cross-cultural research is needed to examine the association between physical function and perceived stress in different ethnic/ racial groups. For clinical implications, health care professionals should be aware that U.S. Chinese older adults with poor physical function may experience high levels of perceived stress. Health care professionals should identify older adults who are vulnerable to stress and provide coping strategies for alleviating psychological distress. In addition, health care professionals should provide educational programs to encourage older adults to engage in regular exercise in order to maintain and/or promote older adults’ physical function and psychological well-being.

## Figures and Tables

**Table 1:** Characteristics of Chinese older adults by the presence of perceived stress (N = 3,116).

Perceived Stress			
	No (n = 122; 3.92%)	Yes (n = 2995; 96.08%)	*p* value
Age (years)	70.35 (± 7.41)	72.80 (± 8.26)	.0013**
Gender			
Male	65 (2.08)	1248 (40.03)	.0141*
Female	57 (1.83)	1748 (56.06)	
Education (years)	9.55 (± 4.45)	8.74 (± 5.05)	.0804
Income (in US$ 5k)	2.23 (±1.47)	1.94 (± 1.12)	.0043**
Living arrangement	2.26 (± 1.93)	1.87 (± 1.90)	.0041**
Years in the US	19.79 (± 13.15)	19.92 (± 13.01)	.9173
Years in the community	13.93 (± 11.60)	12.05 (± 11.01)	.0224*
Medical comorbidities	1.75 (± 1.48)	2.07 (± 1.46)	.0107*
Self-reported Physical Function			
ADL impairment	0.02 (± 0.20)	0.36 (± 1.98)	.0130*
IADL impairment	1.37 (± 3.45)	3.71 (± 6.21)	.0001***
Index of mobility scale	0.33 (± 0.83)	0.72 (± 1.05)	.0001***
Index of basic physical activities scale	1.25 (± 2.09)	3.23 (± 4.04)	.0001***
Directly-observed Physical Function			
Chair stand	3.33 (± 1.39)	2.94 (± 1.53)	.0075**
Tandem stand	4.80 (± 0.74)	4.44 (± 1.74)	.0001***
Timed walk	3.60 (± 1.13)	2.91 (± 1.45)	.0001***
SPPB	11.72 (± 2.31)	10.33 (± 3.24)	.0001***

**Note**: Values are presented as n (%) or mean ± SD. ADL: Katz Activities of Daily Living; IADL: Lawton Instrumental Activities of Daily Living; SPPB: Short Physical Performance Battery

* *p* < 0.05

** *p* < 0.01

*** *p* < 0.001

**Table 2:** Spearman correlations between perceived stress and physical function (N=3,116).

	Perceive Stress	ADL impairment	IADL impairment	Mobility	Physical Activities	Chair Stand	Tandem Stand	Walk	SPPB
Perceived Stress	1.0								
ADL impairment	0.17***	1.0							
IADL impairment	0.28***	0.42***	1.0						
Mobility	0.29***	0.44***	0.62***	1.0					
Physical Activities	0.34***	0.38***	0.55***	0.65***	1.0				
Chair Stand	−0.21***	−0.33***	−0.40***	−0.43***	−0.48***	1.0			
Tandem Stand	−0.18***	−0.43***	−0.45***	−0.43***	−0.43***	0.42***	1.0		
Walk	−0.27***	−0.30***	−0.46***	−0.41***	−0.32***	0.36***	0.42***	1.0	
SPPB	−0.27***	−0.36***	−0.52***	−0.51***	−0.49***	0.81***	0.63***	0.80***	1.0

Note:

* *p* < 0.05

** *p* < 0.01

*** *p* < 0.001

ADL: Katz Activities of Daily Living; IADL: Lawton Instrumental Activities of Daily Living; Mobility: Index of Mobility Scale; Physical Activities: Index of Basic Physical Activities Scale; SPPB: Short Physical Performance Battery

**Table 3:** Self-reported Physical Function on Any Perceived Stress (N = 3,116).

	Model A	Model B	Model C	Model D
	OR (95% CI)			
Age	1.03 (1.01, 1.06)**	1.03 (1.00, 1.05)	1.03 (1.00, 1.06)*	1.03 (1.00, 1.06)
Gender (female)	1.55 (1.07, 2.24)*	1.52 (1.05, 2.21)*	1.52 (1.04, 2.20)*	1.46 (1.00, 2.13)*
Education	0.98 (0.95, 1.02)	0.99 (0.95, 1.03)	0.98 (0.94, 1.02)	0.98 (0.94, 1.02)
Income		0.87 (0.77, 0.98)*	0.90 (0.78, 1.02)	0.90 (0.79, 1.02)
Living arrangement		0.93 (0.85, 1.02)	0.92 (0.83, 1.01)	0.92 (0.84, 1.02)
Years in community			0.97 (0.95, 1.00)*	0.97 (0.95, 1.00)*
Years in U.S.			1.01 (0.99, 1.03)	1.01 (0.98, 1.03)
Medical comorbidities				1.09 (0.95, 1.25)
**ADL impairment**	**1.75 (0.78, 3.91)**	**1.76 (0.78, 3.93)**	**1.80 (0.79, 4.10)**	**1.77 (0.78, 3.99)**
Age	1.02 (0.99, 1.05)	1.01 (0.98, 1.04)	1.02 (0.99, 1.05)	1.01 (0.98, 1.04)
Gender (female)	1.42 (0.98, 2.08)	1.40 (0.96, 2.05)	1.40 (0.96, 2.05)	1.37 (0.93, 2.01)
Education	0.98 (0.94, 1.02)	0.99 (0.95, 1.03)	0.98 (0.94, 1.02)	0.98 (0.94, 1.02)
Income		0.87 (0.78, 0.98)*	0.90 (0.79, 1.03)	0.90 (0.79, 1.03)
Living arrangement		0.92 (0.84, 1.02)	0.91 (0.83, 1.01)	0.91 (0.83, 1.01)
Years in community			0.97 (0.95, 1.00)*	0.97 (0.95, 1.00)*
Years in U.S.			1.01 (0.99, 1.03)	1.01 (0.98, 1.03)
Medical comorbidities				1.07 (0.92, 1.23)
**IADL impairment**	1.11 (1.04, 1.18)**	1.11 (1.04, 1.18)**	1.11 (1.04, 1.18)**	1.10 (1.03, 1.18)**
Age	1.02 (1.00, 1.05)	1.02 (0.99, 1.05)	1.02 (0.99, 1.05)	1.02 (0.99, 1.05)
Gender (female)	1.47 (1.01,2.13)*	1.45 (1.00, 2.11)	1.45 (1.00, 2.12)	1.42 (0.97, 2.07)
Education	0.98 (0.94, 1.02)	0.99 (0.95, 1.02)	0.98 (0.94, 1.02)	0.98 (0.94, 1.02)
Income		0.87 (0.78, 0.98)*	0.90 (0.79, 1.03)	0.90 (0.79, 1.03)
Living arrangement		0.94 (0.85, 1.03)	0.93 (0.84, 1.02)	0.93 (0.84, 1.02)
Years in community			0.98 (0.95, 1.00)*	0.98 (0.95, 1.00)*
Years in U.S.			1.01 (0.98, 1.03)	1.01 (0.98, 1.03)
Medical comorbidities				1.07 (0.93, 1.23)
**Index of mobility scale**	1.46 (1.13, 1.90)**	1.45 (1.11, 1.88)**	1.42 (1.09, 1.85)**	1.39 (1.07, 1.82)*
Age	1.02 (0.99, 1.04)	1.01 (0.98, 1.04)	1.02 (0.99, 1.04)	1.02 (0.99, 1.04)
Gender (female)	1.32 (0.91, 1.93)	1.31 (0.90, 1.93)	1.32 (0.90, 1.94)	1.32 (0.90, 1.93)
Education	0.98 (0.95, 1.02)	0.99 (0.95, 1.03)	0.98 (0.94, 1.02)	0.98 (0.94, 1.02)
Income		0.89 (0.79, 1.00)	0.92 (0.80, 1.05)	0.92 (0.80, 1.05)
Living arrangement		0.94 (0.85, 1.03)	0.93 (0.84, 1.02)	0.93 (0.84, 1.02)
Years in community			0.98 (0.96, 1.00)	0.98 (0.96, 1.00)
Years in U.S.			1.00 (0.98, 1.03)	1.00 (0.98, 1.03)
Medical comorbidities				1.02 (0.88, 1.17)
**Index of basic physical activities scale**	1.21 (1.11, 1.32)***	1.20 (1.10, 1.31)***	1.19 (1.09, 1.30)***	1.19 (1.09, 1.30)***

Note.

* *p* < 0.05

** *p* < 0.01

*** *p* < 0.001

Outcome: Perceived stress was categorized as no stress and any stress. No stress as reference group.

Independent variable: Activities of daily living (ADL) impairment, instrumental activities of daily living (IADL) impairment; index of mobility scale, and index of basic physical activities scale.

**Table 4:** Directly-observed Physical Function on Any Perceived Stress (N = 3,116).

	Model A	Model B	Model C	Model D
	OR (95% CI)			
Age	1.03 (1.01, 1.06)*	1.03 (1.00, 1.05)	1.03 (1.00, 1.06)*	1.03 (1.00, 1.06)
Gender (female)	1.51 (1.04, 2.18)*	1.49 (1.03, 2.17)	1.49 (1.02, 2.17)*	1.44 (0.99, 2.11)
Education	0.98 (0.95, 1.02)	0.99 (0.95, 1.02)	0.98 (0.94, 1.02)	0.98 (0.94, 1.02)
Income		0.88 (0.78, 0.98)	0.90 (0.79, 1.03)	0.90 (0.79, 1.03)
Living arrangement		0.93 (0.85, 1.02)	0.92 (0.84, 1.01)	0.92 (0.84, 1.02)
Years in community			0.97 (0.95, 1.00)*	0.97 (0.95, 1.00)*
Years in U.S.			1.01 (0.98, 1.03)	1.01 (0.98, 1.03)
Medical comorbidities				1.09 (0.95, 1.25)
**Chair stand**	**0.91 (0.79, 1.04)**	**0.92 (0.80, 1.05)**	**0.92 (0.80, 1.05)**	**0.93 (0.81, 1.07)**
Age	1.03 (1.00, 1.05)*	1.02 (0.99, 1.05)	1.02 (1.00, 1.05)	1.02 (0.99, 1.05)
Gender (female)	1.51 (1.04, 2.19)*	1.50 (1.03, 2.18)*	1.49 (1.03, 2.17)*	1.45 (0.99, 2.11)
Education	0.99 (0.95, 1.02)	0.99 (0.95, 1.03)	0.99 (0.95, 1.03)	0.98 (0.95, 1.02)
Income		0.90 (0.77, 0.98)*	0.90 (0.78, 1.02)	0.90 (0.79, 1.02)
Living arrangement		0.94 (0.85, 1.03)	0.92 (0.84, 1.02)	0.93 (0.84, 1.02)
Years in community			0.97 (0.95, 1.00)*	0.97 (0.95, 1.00)*
Years in U.S.			1.01 (0.99, 1.03)	1.01 (0.98, 1.03)
Medical comorbidities				1.08 (0.94, 1.24)
**Tandem stand**	0.71 (0.52, 0.96)*	0.71 (0.53, 0.95)*	0.70 (0.52, 0.94)*	0.71 (0.52, 0.96)*
Age	1.02 (1.00, 1.05)	1.02 (0.99, 1.04)	1.02 (0.99, 1.05)	1.02 (0.99, 1.05)
Gender (female)	1.50 (1.04, 2.18)*	1.50 (1.03, 2.18)*	1.49 (1.02, 2.16)*	1.45 (0.99, 2.11)
Education	1.00 (0.96, 1.04)	1.00 (0.96, 1.04)	1.00 (0.96, 1.04)	1.00 (0.96, 1.04)
Income		0.87 (0.77, 0.98)*	0.91 (0.79, 1.03)	0.91 (0.80, 1.03)
Living arrangement		0.94 (0.85, 1.03)	0.92 (0.84, 1.02)	0.92 (0.84, 1.02)
Years in community			0.97 (0.95, 1.00)*	0.97 (0.95, 1.00)*
Years in U.S.			1.00 (0.98, 1.23)	1.00 (0.98, 1.03)
Medical comorbidities				1.07 (0.93, 1.23)
**Timed walk**	0.75 (0.64, 0.87)***	0.75 (0.64, 0.87)***	0.73 (0.63, 0.85)***	0.73 (0.63, 0.86)***
Age	1.02 (0.99, 1.05)	1.01 (0.98, 1.04)	1.02 (0.99, 1.04)	1.01 (0.99, 1.04)
Gender (female)	1.45 (1.00, 2.10)	1.44 (0.99, 2.09)	1.43 (0.98, 2.08)	1.40 (0.96, 2.04)
Education	0.99 (0.96, 1.03)	1.00 (0.96, 1.04)	1.00 (0.96, 1.04)	0.99 (0.95, 1.03)
Income		0.88 (0.78, 0.98)*	0.91 (0.79, 1.03)	0.91 (0.80, 1.04)
Living arrangement		0.94 (0.85, 1.03)	0.92 (0.84, 1.02)	0.92 (0.84, 1.02)
Years in community			0.97 (0.95, 1.00)*	0.97 (0.95, 1.00)*
Years in U.S.			1.01 (0.98, 1.03)	1.00 (0.98, 1.03)
Medical comorbidities				1.05 (0.92, 1.21)
**SPPB**	0.87 (0.80, 0.94)***	0.87 (0.81,0.95)***	0.87 (0.80, 0.94)***	0.87 (0.80, 0.95)**

Note:

* *p* < 0.05

** *p* < 0.01

*** *p* < 0.001

Outcome: Perceived stress was categorized as no stress and any stress. No stress as reference group.

Independent variable: Chair stand, tandem stand, timed walk, and Short Physical Performance Battery (SPPB).
